# A Matrix Information-Geometric Method for Change-Point Detection of Rigid Body Motion

**DOI:** 10.3390/e21050531

**Published:** 2019-05-25

**Authors:** Xiaomin Duan, Huafei Sun, Xinyu Zhao

**Affiliations:** 1 School of Science, Dalian Jiaotong University, Dalian 116028, China; 2 Beijing Key Laboratory on MCAACI, Beijing Institute of Technology, Beijing 100081, China; 3 School of Materials Science and Engineering, Dalian Jiaotong University, Dalian 116028, China

**Keywords:** change-point detection, special Euclidean group, matrix information geometry, Lie algebra

## Abstract

A matrix information-geometric method was developed to detect the change-points of rigid body motions. Note that the set of all rigid body motions is the special Euclidean group SE(3), so the Riemannian mean based on the Lie group structures of SE(3) reflects the characteristics of change-points. Once a change-point occurs, the distance between the current point and the Riemannian mean of its neighbor points should be a local maximum. A gradient descent algorithm is proposed to calculate the Riemannian mean. Using the Baker–Campbell–Hausdorff formula, the first-order approximation of the Riemannian mean is taken as the initial value of the iterative procedure. The performance of our method was evaluated by numerical examples and manipulator experiments.

The research of capturing motion, including both rotational and translational information, has been investigated by many scholars [1,2,3,4,5,6]. Abrupt changes of time series often contain critically important information, hence the problem of discovering time points where changes occur, called *change points*. Moreover, if we are able to discover all the change points of a discrete time series, then the smooth motion trajectories between each adjacent change point can be obtained. The present paper focuses on change-point detection for a time series of elements in the rigid body motion group. This group is the special Euclidean group in $R^{3}$, denoted by SE(3).

Most of the existing detection techniques apply only to scalar or vector time series [7,8,9,10,11]. Furthermore, some of these methods only discuss very simple models that cannot provide a suitable solution to deal with time series of elements in more complex structures [12,13]. For example, an efficient and robust means method was proposed in [12], but it was performed only by means of linear operations. It is well-known that SE(3) does not satisfy closure under linear combination. Consequently, most of the statistical properties (e.g., the mean) cannot be properly estimated in a straightforward manner. Recently, Merckel and Nishida presented a means method that took advantage of the Lie group structure of SE(3) [14], but their method was constrained by the condition that each displacement $\log(M_{i}^{-1}M_{i+1})$ between two consecutive points $M_{i}$ and $M_{i+1}$ of a series in SE(3) should be small enough. The means method has also been adapted by other scholars [12,15,16]. For example, to improve the detection performance of a mechanical scan radar system, Liu et al. [15] designed a detector based on the Riemannian geometry of Toeplitz covariance matrices. Arnaudon et al. [16] also used the Riemannian geometry of Toeplitz covariance matrices to develop a detection approach for high-resolution Doppler processing. In this paper, we use the means method that, once a change-point occurs, the distance between the current point and the Riemannian mean of its neighbor points should be a local maximum.

Classical information geometry is developed from the research of the statistical manifolds consisting of probability density functions, where the Fisher information matrix was first taken as a Riemannian metric by Rao [17]. Chentsov [18] introduced a family of affine connections and Efron [19] found the relationship between curvatures and statistical inference. Moreover, the theory of dual connections was investigated by Amari [20]. Thus, classical information geometry covers a range of random cases. In order to study non-random cases, Barbaresco, Nielsen, and Bhatia et al. introduced the concept of matrix information geometry, which is mainly used to study radar signal processing, manifold learning, optimization, system stability and optimization, image processing, and so on [21,22]. Especially, matrix Lie groups (including orthogonal group, unitary group, special symplectic group, special Euclidean group, etc.) and submanifolds of the general linear group (including positive definite matrix manifold, Stiefel manifold and Grassman manifold, etc.) have important applications in many fields. Here, the left-invariant (or right-invariant) metric is adopted as the Riemannian metric and the geodetic distance is used to define the distance function. Importantly, the geodesic can be expressed explicitly by exponential map and logarithmic map on manifold, which is very convenient for solving practical problems.

In this paper, a matrix information-geometric method is presented to detect change points, based on the differential-geometric structure of SE(3) and not restricted by the above-mentioned condition in [14]. Note that the SE(3) group is unclosed under linear combination, so we first define the Riemannian mean in view of the Lie group structure of SE(3). Then, a gradient descent algorithm is proposed to calculate the Riemannian mean. Based on the Baker–Campbell–Hausdorff formula, the first-order approximation of the Riemannian mean is taken as the initial value of the iterative procedure so that the algorithm converges rapidly. Simulations and experiments were run to show the computational behavior of the proposed method.

The paper is organized as follows: Section 1 briefly introduces the Riemannian framework of Riemannian manifolds and matrix Lie groups. In Section 2, our proposed change-point detection method is described based on the Lie group structures of SE(3). Section 3 reports the experimental results to demonstrate the effectiveness of our method.

## 1. A Survey of Some Geometrical Concepts

In this section, we give a brief overview of Riemannian manifolds and matrix Lie groups [23,24,25,26,27,28,29], which forms the foundation of our change-point detection method on SE(3).

### 1.1. Riemannian Manifolds

Let us denote a Riemannian manifold by M. Its tangent space at point X∈M is denoted by $T_{X}M$. Given any pair of points $U,V\in T_{Y}M$, an inner product ${\langle U,V\rangle }_{X}\in R$ can be defined. Let γ:[0,1]→M be a sufficiently smooth curve on M, then the length of γ(t) is defined by

(1) $$\ell \left(\gamma \right):=\int_{0}^{1}\sqrt{{\langle \dot{\gamma }\left(t\right),\dot{\gamma }\left(t\right)\rangle }_{\gamma (t)}}dt=\int_{0}^{1}\sqrt{\mathrm{tr}[{\left(\gamma {\left(t\right)}^{-1}\dot{\gamma }\left(t\right)\right)}^{T}\gamma {\left(t\right)}^{-1}\dot{\gamma }\left(t\right)]}dt,$$

where tr denotes the trace of a matrix. The geodesic distance between two matrices *X* and *Y* on M is the infimum of the lengths of all curves connecting them

(2) d(X,Y):=inf{ℓ(γ)∣γ:[0,1]→Mwithγ(0)=X,γ(1)=Y},

which can be achieved by the length of geodesics connecting them.

Given a regular function f:M→R, its Riemannian gradient $\nabla_{X}f$ in the direction of the vector $V\in T_{X}M$ measures the change rate of the function *f* in the direction *V*. Namely, for any smooth curve ϕ:[0,1]→M satisfying ϕ(0)=X∈M and $\dot{\phi }\left(0\right)=V$, the Riemannian gradient $\nabla_{X}f$ is the unique vector in $T_{X}M$ such that

(3) $${\langle V,\nabla_{X}f\rangle }_{X}=\frac{d}{dt}f\left(\phi \left(t\right)\right){|}_{t=0}.$$

### 1.2. Matrix Lie Groups

A group *G* is called a Lie group if it has differentiable structure, such that the group operators

(4) $$\begin{array}{c} \mu :G\times G\to G,\;\;\;\;(X,Y)\mapsto X\cdot Y\\ \iota :G\to G,\;\;\;\;\;X\mapsto X^{-1} \end{array}$$

are differentiable, X,Y∈G. A matrix Lie group is a Lie group consisting of matrices. The tangent space of *G* at the identity is the Lie algebra g, where the Lie bracket is defined.

It is essential that the information between *G* and g is transmitted by exponential map and logarithmic map. The map from g to *G* is the exponential map, denoted by exp. In most problems, the exponential map has the characteristics of neither injective nor surjective projection, but it is a homeomorphic mapping from a neighborhood of the identity I∈G to a neighborhood of the identity 0∈g. On the other hand, there is a map from *G* to g, called the logarithmic map, denoted by log, which is the local inverse of the exponential map.

It is well known that the exponential function has the property exp(a)exp(b)=exp(a+b) with real numbers *a* and *b*, but there is no such property in non-commutative Lie groups, such as the special orthogonal group. The product in logarithmic coordinates is defined as a mapping ν:g×g→g such that

exp(U)exp(V)=exp(ν(U,V)),

where the Baker–Campbell–Hausdorff formula

(5) $$\nu \left(U,V\right)=U+V+\left[U,V\right]+O(|\left(U,V\right){|}^{3})$$

represents the Taylor series for ν(U,V) about the point (0,0).

The set of n×n real matrices is denoted by $M_{n\times n}$ and the set consisting of all invertible n×n real matrices is called the general linear group, denoted by GL(n,R). Moreover, using the continuous map X↦det(X), GL(n,R) is an open subset of $M_{n\times n}$ and isomorphic to $R^{n\times n}$. Hence, GL(n,R) is a differentiable manifold.

On GL(n,R), the group multiplication is defined as the matrix multiplication. Its inverse map is a matrix *X* on GL(n,R) to its inverse matrix $X^{-1}$, and the identity element on GL(n,R) is the identity matrix *I*. In addition, the Lie algebra gl(n,R) of GL(n,R) is $M_{n\times n}$ with the Lie bracket as follows:

(6) [U,V]=UV−VU,∀U,V∈gl(n,R).

All other real matrix Lie groups are subgroups of GL(n,R). At the same time, their group operators are subgroup restriction of the one on GL(n,R). The Lie bracket on their Lie algebras is the matrix commutator.

Let *S* and s represent a matrix Lie group and its Lie algebra, respectively. Then, the exponential map of s is the exponential of the matrix. That is to say, if an element V∈s is given, the exponential map is as follows:

(7) $$\exp\left(V\right)=\sum_{m=0}^{\infty }\frac{V^{m}}{m!}.$$

The definition of the logarithmic map is as follows:

(8) $$\log\left(X\right)=\sum_{m=1}^{\infty }{\left(-1\right)}^{m+1}\frac{{\left(X-I\right)}^{m}}{m},$$

where *X* belongs to a neighborhood of the identity *I* on *S*.

A matrix Lie group also has the geometric structure of manifolds. Let X∈S, the tangent space of *S* at *X* is denoted by $T_{X}S$. Then for any X,Y∈S and $V\in T_{X}S$, we have the following maps

(9) $$\begin{array}{c} L_{X}Y=XY,\;\;{\left(L_{X}\right)}_{*}V=XV,\\ R_{X}Y=YX^{-1},\;\;{\left(R_{X^{-1}}\right)}_{*}V=VX, \end{array}$$

where *L* and *R* respectively denote the left translation and the right translation, and ${\left(•\right)}_{*}$ represents the tangent mapping of •. The adjoint action $Ad_{A}:s\to s$ is defined as

(10) $$Ad_{A}X=AXA^{-1}.$$

From (9) and (10), the following formula is easy to obtain:

(11) $$Ad_{A}=L_{A}R_{A}.$$

Therefore, for X∈S and $U,V\in T_{X}S$, we obtain the left invariant metric on *S* as follows:

(12) $${\langle U,V\rangle }_{X}={\langle {\left(L_{X^{-1}}\right)}_{*}U,{\left(L_{X^{-1}}\right)}_{*}V\rangle }_{I}=\langle X^{-1}U,X^{-1}V)\rangle_{I}:=\mathrm{tr}\left[{\left(X^{-1}U\right)}^{T}X^{-1}V\right].$$

### 1.3. Special Euclidean Group

Let SO(n) denote the special orthogonal group acting on $R^{n}$, then the special Euclidean group SE(n) is represented as

(13) $$SE\left(n\right)=\left\{\left(\begin{array}{cc} A & b\\ 0 & 1 \end{array}\right)\;|\;A\in SO\left(n\right),b\in R^{n}\right\}.$$

It is well-known that the action of SE(n) on $R^{n}$ is equivalent to a rotation with a translation, namely, the semi-direct product of SO(n) with $R^{n}$ [30], as follows:

(14) $$SE\left(n\right)=SO\left(n\right)⋉R^{n}.$$

The Lie algebra se(n) of SE(n) can be expressed by

(15) $$\mathrm{se}\left(n\right)=\left\{\left(\begin{array}{cc} \Omega & v\\ 0 & 0 \end{array}\right)\;|\;\Omega^{T}=-\Omega ,v\in R^{n}\right\}.$$

The tangent space $T_{X}SE\left(n\right)$ and the normal space $N_{X}SE\left(n\right)$ associated with SE(n) can be characterized as follows:

(16) $$T_{X}SE\left(n\right)=\left\{XU|U\in \mathrm{se}\left(n\right)\right\},$$

(17) $$N_{X}SE\left(n\right)=\left\{{\left(X^{-1}\right)}^{T}\left(\begin{array}{c} \begin{array}{cc} s & 0\\ h & d \end{array} \end{array}\right)|X\in R^{n\times n},s^{T}=s,h\in R^{1\times n},d\in R\right\}.$$

Notice that SE(n) is connected, which means that for any given pair X,Y, we can find a geodesic

(18) $$\gamma \left(t\right)=X\exp(t\log\left(X^{-1}Y\right))$$

such that γ(0)=X and γ(1)=Y, by taking the initial velocity as $\dot{\gamma }\left(0\right)=\log\left(X^{-1}Y\right)\in \mathrm{se}\left(n\right)$.

When n=3, a matrix of SE(3) represents a displacement of the rigid body, where *A* denotes the orientation or attitude and *b* corresponds to the translation. The skew-symmetric matrix Ω in se(3) can be uniquely written as

(19) $$\Omega =\left(\begin{array}{ccc} 0 & -\omega_{z} & \omega_{y}\\ \omega_{z} & 0 & -\omega_{x}\\ -\omega_{y} & \omega_{x} & 0 \end{array}\right),$$

with $\omega =\left(\omega_{x},\omega_{y},\omega_{z}\right)\in R^{3}$. Let ${∥\cdot ∥}_{F}$ denote the Frobenius norm, then ${∥\omega ∥}_{F}$ expresses the amount of rotation corresponding to the identity vector along ω. Physically, ω stands for the angular velocity of the rigid body motion and *v* represents its linear velocity [31].

## 2. Proposed Method for Change-Point Detection

### 2.1. Design for Change-Point Detection Method

Let $\left(X_{1},\ldots ,X_{n}\right)$ denote a time series. The detection procedure is formulated as follows: For each point $X_{i}$ under test, the Riemannian mean of the 2N+1 points from $X_{i-N}$ to $X_{i+N}$ is calculated. Here *N* is the window size and its value can be selected experimentally. Then, the Riemannian distance $d_{i}$ from $X_{i}$ to its Riemannian mean of $X_{i}$ can be obtained. If this distance $d_{i}$ is a local maximum, then $X_{i}$ is possibly a change point.

Substituting (18) into (1), the Riemannian distance between *X* and *Y* on SE(3), which arises from the left-invariant metric (12), is expressed as

(20) $$d\left(X,Y\right)=∥\log\left(X^{-1}Y\right){∥}_{F}.$$

Thus, for each point $X_{i}$, the Riemannian mean $M_{f}\left(X_{i}\right)$ of 2N+1 points from $X_{i-N}$ to $X_{i+N}$ is the point *X* minimizing

(21) $$f\left(X\right)=\frac{1}{2(2N+1)}\sum_{j=i-N}^{i+N}d{\left(X,X_{j}\right)}^{2}.$$

**Figure 1:**
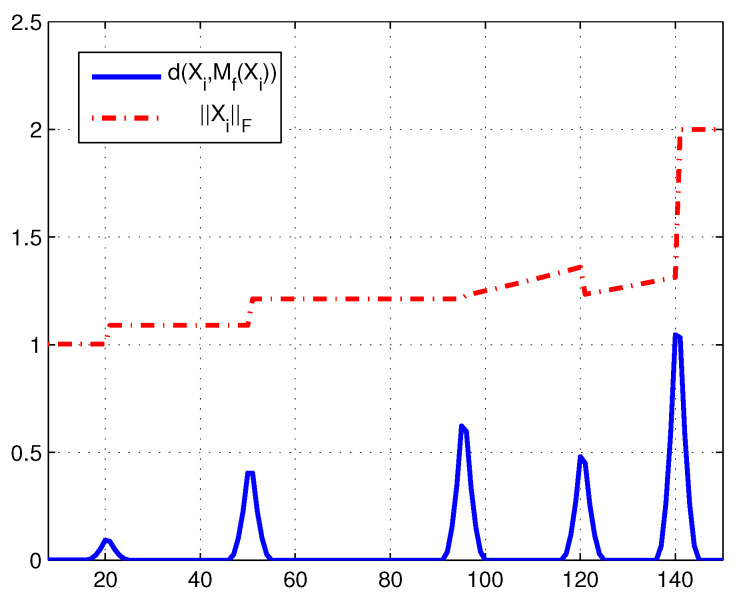
When a change-point occurs, the series ${\left(d\left(X_{i},M_{f}\left(X_{i}\right)\right)\right)}_{i}$ displays a local maximum.

Then, our principle can be redescribed as that $X_{i}$ is found to possibly be a change point if $d(X_{i},M_{f}\left(X_{i}\right))$ is a local maximum of the Riemannian distances ${\left(d\left(X_{i},M_{f}\left(X_{i}\right)\right)\right)}_{i}$. As Figure 1 shows, whenever a change-point appears, the series ${\left(d\left(X_{i},M_{f}\left(X_{i}\right)\right)\right)}_{i}$ displays a local maximum point.

### 2.2. Method for Change-Point Detection

As previously discussed, this subsection proposes an algorithm to calculate the series ${\left(d\left(X_{i},M_{f}\left(X_{i}\right)\right)\right)}_{i}$ so that all local maximum points may be sought out. Actually, Riemannian means in Lie group have been discussed well in [27,32]. Here these ideas are extended to propose an algorithm for calculating the Riemannian mean of a set of 2N+1 points on SE(3).

First, we need to compute the Riemannian gradient of the objective function f(X) to be optimized. In the definition of the Riemannian gradient (3), the generic smooth curve ϕ may be replaced with a geodesic, then we obtain the following theorem.

**Theorem** **1.** *The Riemannian gradient of the objective function* f(X) *at point X is given by*

(22) $$\nabla_{X}f\left(X\right)=\frac{1}{2N+1}X\sum_{j=i-N}^{i+N}\log\left(X_{j}^{-1}X\right).$$

**Proof.**  Let s(t)=Xexp(tV) denote the geodesic emanating from *X* in the direction $V=\dot{s}\left(0\right)$, then the real-valued function is written as

(23) $$f\left(s(t)\right)=\frac{1}{2(2N+1)}\sum_{j=i-N}^{i+N}\mathrm{tr}\left[\log{\left(X_{j}^{-1}s\left(t\right)\right)}^{T}\log\left(X_{j}^{-1}s\left(t\right)\right)\right].$$

Using Proposition 2.1 in [33] and some properties of the trace, we have the derivative of $f\left(s(t)\right)$ with respect to *t* as follows:

(24) $$\frac{d}{dt}f\left(s(t)\right){|}_{t=0}=\frac{1}{2N+1}\sum_{j=i-N}^{i+N}\mathrm{tr}\left[\log{\left(X_{j}^{-1}X\right)}^{T}X^{-1}V\right]={\langle \frac{1}{2N+1}X\sum_{j=i-N}^{i+N}\log\left(X_{j}^{-1}X\right),V\rangle }_{X}.$$

From (3), it is shown that Formula (22) is valid. □

Let $\exp_{X}$ denote the Riemannian exponential map about the point *X*, then the Riemannian gradient algorithm can be expressed as [23,24]:

(25) $$X:=\exp_{X}\left(-\nabla_{X}f\left(X\right)\right).$$

From (16), the Riemannian exponential map expX on SE(n) is defined by $\exp_{X}\left(XA\right)=X\exp\left(A\right)$. By Theorem 1, the Riemannian gradient algorithm for calculating the Riemannian mean $M_{f}\left(X_{i}\right)$ of 2N+1 points from $X_{i-N}$ to $X_{i+N}$ is rewritten as

(26) $$X:=X\exp(\frac{1}{2N+1}\sum_{j=i-N}^{i+N}\log\left(X^{-1}X_{j}\right)).$$

According to (22), the Riemannian mean is the stable point of Algorithm (26) if and only if

(27) $$\sum_{j=i-N}^{i+N}\log\left(X^{-1}X_{j}\right)=0.$$

As algorithm (26) converges rapidly to the Riemannian mean $M_{f}\left(X_{i}\right)$, the first-order approximate value of $M_{f}\left(X_{i}\right)$ is taken as the initial value of the iterative procedure. In fact, using the Baker–Campbell–Hausdorff formula (5) up to first-order terms, the Riemannian distance (20) between two points on SE(3) can be approximated as follows:

(28) $$d\left(X,Y\right)\approx {∥\log Y-\log X∥}_{F}.$$

Here the sum of squared approximate distances in (21) is minimized by the arithmetic mean of the logarithmic maps of the points in the neighborhood of $X_{i}$, so the first-order approximation of the Riemannian mean $M_{f}\left(X_{i}\right)$ is given by

(29) $${\hat{X}}_{i}=\exp\left(\frac{1}{2N+1}\sum_{j=i-N}^{i+N}\log\left(X_{j}\right)\right).$$

To sum up, the algorithm for calculating the series ${\left(d\left(X_{i},M_{f}\left(X_{i}\right)\right)\right)}_{i}$, in which the first-order approximation of the Riemannian mean is taken the initial value, is presented as follows:

**Algorithm 1** Algorithm for calculating the series of geodesic distances**Inputs:** Points $X_{1},\ldots ,X_{n}$ on SE(3) and window size *N*. **Output:** The approximation of the series ${\left(d\left(X_{i},M_{f}\left(X_{i}\right)\right)\right)}_{i}$. **For**
i=N+1,…,n−N. **Initialization:** Set ${\hat{X}}_{i}:=\exp\left(\frac{1}{2N+1}\sum_{j=i-N}^{i+N}\log\left(X_{j}\right)\right).$ **Main loop:** $dX_{j}={\hat{X}}_{i}^{-1}X_{j},j=i-N,\ldots ,i,\ldots ,i+N$. $d{\hat{X}}_{i}=\exp\left(\frac{1}{2N+1}\sum_{j=i-N}^{i+N}\log\left(dX_{j}\right)\right)$. ${\hat{X}}_{i}={\hat{X}}_{i}d{\hat{X}}_{i}$. If $∥\sum_{j=i-N}^{i+N}\log\left(dX_{j}\right){∥}_{F}$ is sufficiently small, the output is $d\left(X_{i},M_{f}\left(X_{i}\right)\right)\approx {∥\log\left(X_{i}\right)-\log\left({\hat{X}}_{i}\right)∥}_{F}$. **Otherwise continue looping.** **Endfor.**

Geometrically, we left-multiply these points by ${\hat{X}}_{i}^{-1}$, so that ${\hat{X}}_{i}^{-1}X_{j}$ (j=i−N,…,i,…,i+N) are moved to the vicinity of the identity. Although this algorithm is proposed to detect the change-points of rigid body motion on SE(3), we can extend it to similar problems on other matrix Lie groups, especially the subgroups of the general linear group.

## 3. Simulations

In this section, we are devoted to illustrating the effectiveness of Algorithm 1. First, the simulation of a cylinder motion is provided, and the effects of the window size *N* are analyzed for the results of the change-point detection. Secondly, the change-point detection experiment is presented, which was carried out using a STAUBERT TX90XL manipulator (Staubli, Faverges, France). Change points of the manipulator’s motion were identified in order to reduce its speed near the change points, so that the manipulator could move smoothly.

### 3.1. Change-Point Detection for the Motion of a Cylinder

**Figure 2:**
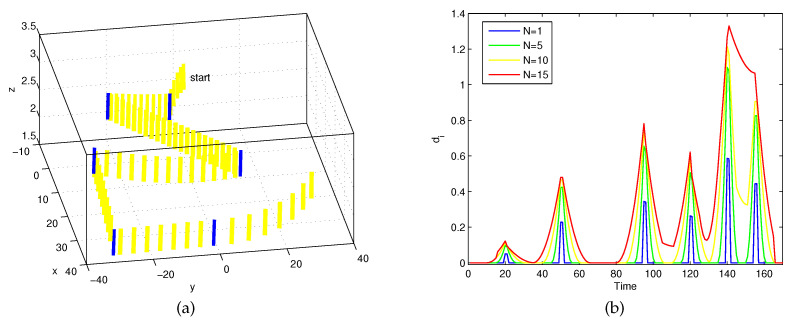
(**a**) Blue cylinders denote identified change-points; (**b**) Detection results with different window sizes.

Consider a cylinder’s synthetic motion $\left(X_{1},\ldots ,X_{200}\right)$, where six change points happen at moments 21, 51, 96, 121, 141, and 156. In Figure 2a, the blue cylinders represent the found change points, with a visual representation of this cylinder at every two poses of the corresponding position $X_{i}$.

The proposed Algorithm 1 was applied to calculate the series ${\left(d\left(X_{i},M_{f}\left(X_{i}\right)\right)\right)}_{i}$ with different window sizes N=1,5,10,15. As Figure 2b shows, a visual representation of the series ${\left(d\left(X_{i},M_{f}\left(X_{i}\right)\right)\right)}_{i}$ is given by four-color simulation curves. Six local maxima were present at indices 21, 51, 96, 121, 141, and 156, so the simulation coincided with the practical motion of the cylinder extremity. Moreover, it can be observed that when the window size N=15, the sixth change-point was missed. Therefore, the window size should not be greater than the difference between the corresponding moments of two adjacent change-points.

### 3.2. Change-Point Detection for the Motion of the STAUBERT TX90XL Manipulator

**Figure 3:**
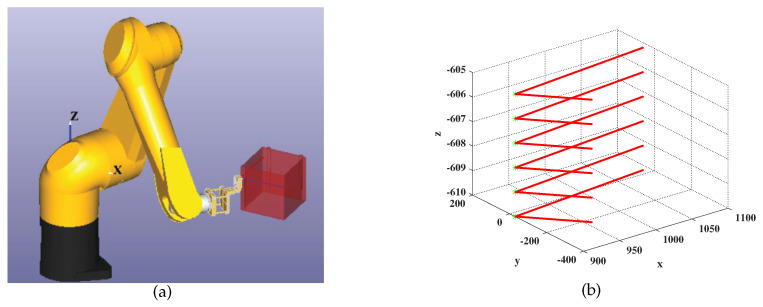
(**a**) Scanning on the surface of a rectangular workpiece with rounded corners by a STAUBLI TX90XL manipulator; (**b**) Change points of the motion trajectory.

The STAUBLI manipulator has high motion accuracy and protection level, which is suitable for welding or ultrasonic non-destructive testing. In our experiment, a STAUBLI TX90XL manipulator is used to scan the surface of a rectangular workpiece with rounded corners, as shown in Figure 3a. The motion trajectory contained 2400 points with 1 mm equal arc length interval, and the tip-position of the flexible manipulator needed to scan back and forth across the rounded rectangular workpiece. Our provided method was used to find the change points, as shown in Figure 3b. After identifying those change points, it was convenient and necessary to reduce the speed of the manipulator at each change-point from 60 mm/s to 30 mm/s, so that a smooth movement could be maintained.

**Figure 4:**
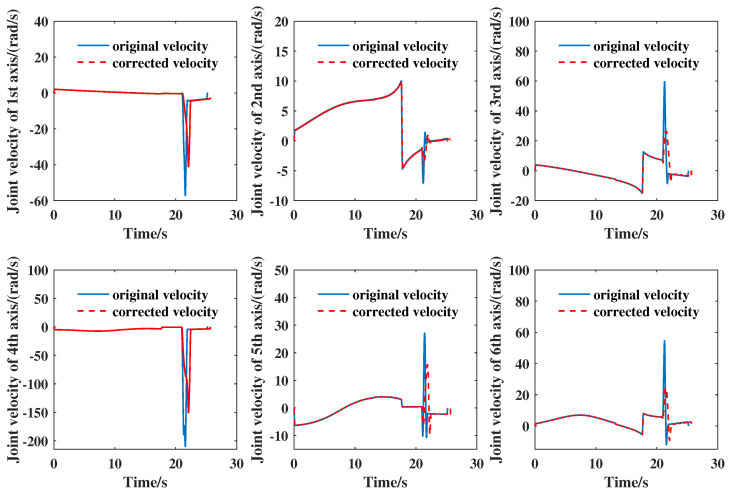
Comparison of the original velocity without change-point detection to the correction velocity for each joint axis.

In the practical experiments, each joint velocity was measured based on the data given by each motor encoder. Figure 4 shows a comparison of the original velocity without change-point detection to the corrected velocity for each joint axis. It can be seen that the peak velocity after change-point detection and calibration was significantly reduced. The maximum velocities of joints 2–3 and 5–6 decreased by more than 40%. This application had good performance in smooth transition and stable operation for the manipulator’s control.

## 4. Conclusions

In this paper, we propose a change-point detection method in the view of matrix information geometry. Using the Lie group structure of SE(3), a gradient descent algorithm is proposed to calculate the Riemannian mean. Based on the Baker–Campbell–Hausdorff formula, the first-order approximation of the Riemannian mean is taken as the initial value so that the iterative procedure can converge rapidly. The numerical example indicates that the provided method can identify these change points with significant posture changes in the rigid body motion. An experiment was also carried out on the motion control of the manipulator. By identifying the change points and reducing the speed of neighbor points, the sharp velocity change of each manipulator joint could be effectively improved, and a smooth transition could be maintained.
